# MicroRNA-335-5p is a potential suppressor of metastasis and invasion in gastric cancer

**DOI:** 10.1186/s13148-017-0413-8

**Published:** 2017-10-17

**Authors:** Alejandra Sandoval-Bórquez, Iva Polakovicova, Nicolás Carrasco-Véliz, Lorena Lobos-González, Ismael Riquelme, Gonzalo Carrasco-Avino, Carolina Bizama, Enrique Norero, Gareth I. Owen, Juan C. Roa, Alejandro H. Corvalán

**Affiliations:** 1 Advanced Center for Chronic Diseases (ACCDiS), Pontificia Universidad Católica de Chile, Santiago, Chile; 2 Laboratory of Molecular Pathology, Department of Pathology, School of Medicine, BIOREN-CEGIN, and Graduate Program in Applied Cell and Molecular Biology, Universidad de La Frontera, Temuco, Chile; 3 Center UC for Investigational in Oncology (CITO), Pontificia Universidad Católica de Chile, Santiago, Chile; 4 Instituto de Química, Faculty of Science, Pontificia Universidad Católica de Valparaíso, Valparaiso, Chile; 5 Advanced Center for Chronic Diseases (ACCDiS), Universidad de Chile, Santiago, Chile; 6 Fundación Ciencia y Vida, Parque Biotecnológico, Santiago, Chile; 7 Department of Pathology, Faculty of Medicine, Hospital Clínico Universidad de Chile, Santiago, Chile; 8 Department of Pathology, Faculty of Medicine, Pontificia Universidad Católica de Chile, Santiago, Chile; 9 Esophagogastric Surgery Unit, Hospital Dr. Sótero del Río, Santiago, Chile; 10 Digestive Surgery Department, Pontificia Universidad Católica de Chile, Santiago, Chile; 11 Department of Physiology, Faculty of Biological Sciences, Pontificia Universidad Católica de Chile, Santiago, Chile; 12 Department of Hematology-Oncology, Faculty of Medicine, Pontificia Universidad Católica de Chile, Santiago, Chile

**Keywords:** miR-335, Gastric cancer, Metastasis, PLAUR, CDH11, Methylation

## Abstract

**Background:**

Multiple aberrant microRNA expression has been reported in gastric cancer. Among them, microRNA-335-5p (miR-335), a microRNA regulated by DNA methylation, has been reported to possess both tumor suppressor and tumor promoter activities.

**Results:**

Herein, we show that miR-335 levels are reduced in gastric cancer and significantly associate with lymph node metastasis, depth of tumor invasion, and ultimately poor patient survival in a cohort of Amerindian/Hispanic patients. In two gastric cancer cell lines AGS and, Hs 746T the exogenous miR-335 decreases migration, invasion, viability, and anchorage-independent cell growth capacities. Performing a PCR array on cells transfected with miR-335, 19 (30.6%) out of 62 genes involved in metastasis and tumor invasion showed decreased transcription levels. Network enrichment analysis narrowed these genes to nine (PLAUR, CDH11, COL4A2, CTGF, CTSK, MMP7, PDGFA, TIMP1, and TIMP2). Elevated levels of PLAUR, a validated target gene, and CDH11 were confirmed in tumors with low expression of miR-335. The 3′UTR of CDH11 was identified to be directly targeted by miR-335. Downregulation of miR-335 was also demonstrated in plasma samples from gastric cancer patients and inversely correlated with DNA methylation of promoter region (Z = 1.96, *p* = 0.029). DNA methylation, evaluated by methylation-specific PCR assay, was found in plasma from 23 (56.1%) out of 41 gastric cancer patients but in only 9 (30%) out of 30 healthy donors (*p* = 0.029, Pearson’s correlation). Taken in consideration, our results of the association with depth of invasion, lymph node metastasis, and poor prognosis together with functional assays on cell migration, invasion, and tumorigenicity are in accordance with the downregulation of miR-335 in gastric cancer.

**Conclusions:**

Comprehensive evaluation of metastasis and invasion pathway identified a subset of associated genes and confirmed PLAUR and CDH11, both targets of miR-335, to be overexpressed in gastric cancer tissues. DNA methylation of miR-335 may be a promissory strategy for non-invasive approach to gastric cancer.

**Electronic supplementary material:**

The online version of this article (10.1186/s13148-017-0413-8) contains supplementary material, which is available to authorized users.

## Background

Gastric cancer is the fifth most common cancer and the third leading cause of cancer-related death in both sexes worldwide with 723,000 deaths reported in 2012 [1]. Gastric cancer presents a wide variation in incidence and mortality rates across different geographical areas suggesting that the development and progression of this disease may be affected by the environment [2]. Although comprehensive studies have identified several genes associated with the development and progression of gastric cancer, the detailed molecular mechanisms of the disease still remain poorly understood [3, 4].

MicroRNAs (miRNAs) are a class of short noncoding RNAs that suppress gene expression resulting in translational repression or mRNA decay [5]. Emerging literature highlights the role of miRNAs in a variety of biological processes within the tumor cell, including proliferation, differentiation, migration, and invasion [6, 7]. Multiple aberrant miRNA expression has also been frequently reported in human neoplasms including gastric cancer [5, 8, 9]. Among relevant miRNAs in cancer, microRNA-335-5p (miR-335) is a transcript of genomic region chromosome 7q32.2, regulated by DNA methylation that has been reported to possess both tumor suppressor and tumor promoter activities depending on the tumor type and stage [10]. However, in gastric cancer, the role of the miRNA has to be defined, as studies have reported both overexpression and downregulation [11, 12].

Here we present clinical and functional assays that suggest that miR-335 is a tumor suppressor in gastric cancer. In addition, we comprehensively evaluate the impact of miR-355 upon metastasis and tumor invasion pathways. Finally, we explore if the downregulation of miR-335 associated with DNA methylation in plasma could be a useful approach to non-invasive assessment of gastric cancer.

## Results

### miR-335 is downregulated in gastric cancer tissues and associated with depth of tumor invasion and lymph node metastasis

**Fig. 1:**
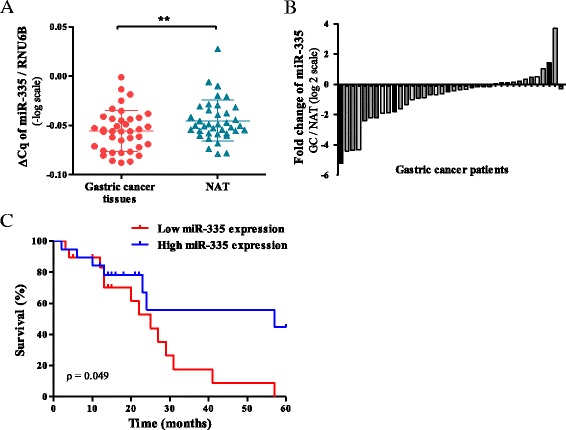
miR-335-5 is downregulated in gastric cancer and associated with poor prognosis. **a** Significant downregulation of miR-335 expression among 38 gastric cancer tissues and their matched non-tumor adjacent tissues (NAT). miR-335 expression was normalized by RNU6. Data were transformed to logarithmic values (−log). Results indicate the mean ± SD. **b** Relative expression level of miR-335 in gastric cancer tissues compared to their NAT. Low expression level of miR-335 was observed in 27 cases. Data were transformed to log2 values. **c** Kaplan-Meier curves of survival time for patients with gastric cancer divided according to low/high miR-335 expression

miR-335 expression was examined in 38 Amerindian/Hispanic advanced gastric cancer tissues and their matched non-tumor adjacent tissues (NAT), showing a significantly reduced expression in tumors (*p* = 0.002), (Fig. 1a, b). Relative expression of miR-335 in tumor samples and its association with clinicopathological features demonstrated a significant negative correlation with depth of tumor invasion (T2 + T3 vs T4, invasion to subserosa group and invasion to serosa group, respectively [13]; *p* = 0.025) and lymph node invasion (*p* = 0.038) according to univariate analysis (Table 1). No correlations between miR-335 expression and other clinicopathological features were found.

**Table 1:** Association of expression levels of miR-335 with clinicopathological features in patients with gastric cancer

Characteristics	Univariate analysis		
	*N*	miR-335 expression (mean ± SD)	*p* value
Gender			0.531
Female	15	0.808 ± 0.515	
Male	23	1.246 ± 2.657	
Age (years)			0.815
< 69	19	0.810 ± 0.621	
≥ 69	19	1.337 ± 2.899	
Tumor location			0.107
Proximal	9	0.987 ± 0.539	
Distal	29	1.100 ± 2.378	
Bormann classification			0.148
I + II	18	1.580 ± 2.962	
III + IV	20	0.618 ± 0.389	
WHO classification			0.188
Tubular	22	0.638 ± 0.399	
Mixed^a^	16	1.672 ± 3.136	
Histological grade			0.564
Poor	16	0.655 ± 0.303	
Moderate	22	1.378 ± 2.713	
Lauren classification			0.081
Intestinal	26	1.104 ± 2.504	
Diffuse	12	1.008 ± 0.606	
Depth of tumor invasion			0.025*
T2 + T3	19	1.602 ± 2.865	
T4	19	0.545 ± 0.368	
Lymph node metastasis			0.038*
N0	11	2.060 ± 3.714	
N1 + N2 + N3	27	0.672 ± 0.549	
Lymphatic venous and perineural invasion			0.410
Negative	21	1.355 ± 2.752	
Positive	17	0.725 ± 0.593	

^a^Papillary/mucinous/signet-ring

*p < 0.05, depth of tumor invasion; lymph node metastasis

### Downregulation of miR-335 is associated with poor prognosis in patients with gastric cancer

We performed overall survival analysis of patients with gastric cancer using Kaplan-Meier analysis according to levels of miR-335 expression (median of the total samples). During a follow-up of 60 months, 20 (52.6%) of the 38 patients died. The median overall survival time was 27 months. Univariate analysis revealed that patients with low expression level of miR-335 had a significantly reduced median overall survival (*p* = 0.049) (Fig. 1c).

### miR-335 suppresses gastric cancer cell migration and invasion in vitro

**Fig. 2:**
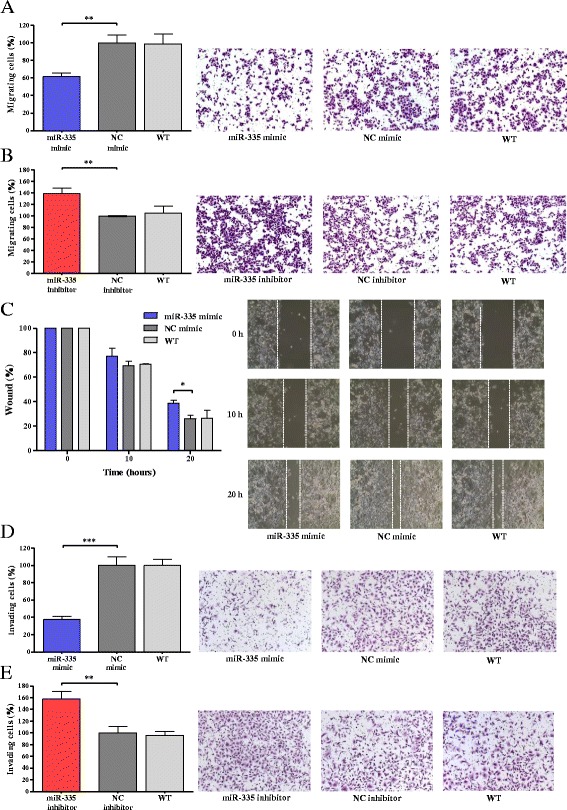
Overexpression of miR-335 inhibits cell migration and invasion. **a**, **b** Representative images of AGS cells transfected with NC/miR-335 mimic or with NC/miR-335 inhibitor in migration assay. **c** Representative images of AGS cells transfected with control NC/miR-335 mimic in wound healing assay. **d**, **e** Representative images of AGS cells transfected with control NC/miR-335 mimic or with NC/miR-335 inhibitor in invasion assay. Results represent the means of three independent experiments, bars indicate SD. *p < 0.05, **p < 0.01, ***p < 0.001. WT, wild-type

To evaluate the functional effect of miR-335 on migration, we selected AGS cells based on expression levels of miR-335 in non-Asian gastric cancer lines (Additional file 1: Figure S1). Cells were transfected with miR-335 mimic or negative control (NC) mimic and miR-335 inhibitor or NC inhibitor, respectively. The results showed that the percentage of cells that migrated through the Transwell was significantly lower in cells with miR-335 mimic (61.7% ± 3.4; *p* < 0.01) than cells transfected with NC mimic (Fig. 2a). In accordance, the number of cells that migrated through the Transwell was significantly higher in cells with miR-335 inhibitor (139.1% ± 9.6; *p* < 0.01) than NC inhibitor (Fig. 2b). There were no significant differences between cells transfected with NCs (mimic and inhibitor) and wild-type (non-transfected) cells.

Migration of cells transfected with miR-335 mimic also was tested in an in vitro wound-healing model. Wound closure was quantified at 0, 10, and 20 h with 0 h set as 100%. The results showed that wound closure was significantly lower in cells treated with miR-335 mimic (38.9% ± 2.2 remaining open; *p* < 0.05) compared to cells treated with the corresponding NC mimic (26.3% ± 2.7) and wild-type cells (26.7% ± 6.3) at 20 h (Fig. 2c), suggesting lower migration of cells expressing elevated levels of miR-335. As expected, there were no significant differences between cells transfected with NC mimic and wild-type cells.

To assess the effect of miR-335 on invasion, the same system as described above was modified to use Transwell inserts coated with Matrigel. We observed that the percentage of cells invading the Matrigel and passing through the Transwell was significantly lower in cells transfected with miR-335 mimic (37.7% ± 3.5; *р* < 0.001) than in cells transfected with NC mimic (Fig. 2d). Accordingly, the percentage of invading cells was significantly higher in cells transfected with miR-335 inhibitor (157.7% ± 12.6; *p* < 0.01) as opposed to cells transfected with the NC inhibitor (Fig. 2e). Again, there were no significant differences between wild-type cells and those transfected with NCs.

### miR-335 reduces tumorigenicity and cell viability in vitro

**Fig. 3:**
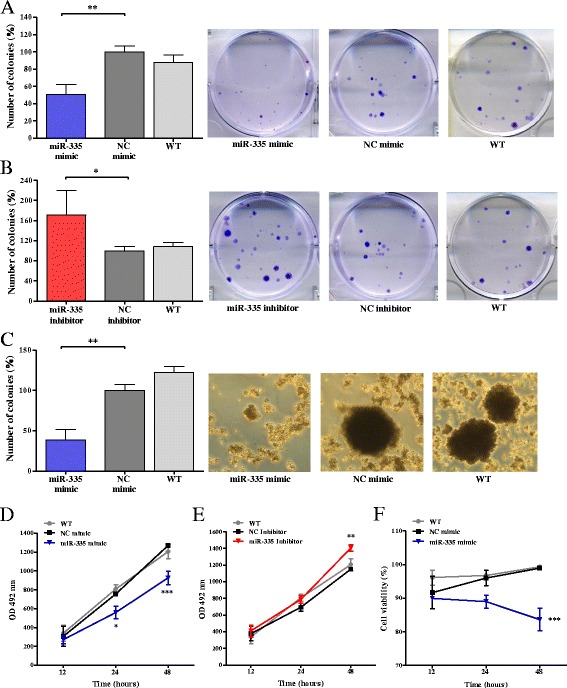
Overexpression of miR-335 reduces clonogenic capacity, anchorage-independent cell growth and cell viability. **a**, **b** Representative images for AGS cells transfected with control NC/miR-335 mimic or with NC/miR-335 inhibitor in clonogenic assay. Crystal violet-stained colonies are shown. **c** AGS cells transfected with control NC/miR-335 mimic in soft agar assay. Formation of colonies > 50 μm in diameter was scored at 3 weeks. **d**, **e** Viability of AGS cells transfected with control NC/miR-335 mimic or with NC/miR-335 inhibitor in MTS assay. **f** Exogenous expression of miR-335 in AGS cells in trypan blue dye exclusion assay. Results represent the means of three independent experiments, bars indicate SD. *p < 0.05, **p < 0.01, ***p < 0.001. WT, wild-type

To further extend the role of miR-335 on migration and invasion in gastric cancer, the effect of miR-335 on clonogenic capacity was determined in cells transfected with either miR-335 mimic or inhibitor. The treated cells were incubated for 14 days to allow colony formation. The results revealed that colony numbers decreased significantly in cells transfected with miR-335 mimic (50.8% ± 11.9; *p* < 0.01) compared to cells transfected with NC mimic (Fig. 3a). Correspondingly, colony formation was significantly increased in cells transfected with miR-335 inhibitor (171% ± 24.3; *p* < 0.05) (Fig. 3b). No statistically significant differences between cells transfected with NCs and wild-type cells were found.

To mimic 3D cellular environment to that seen in vivo, we performed the soft agar assay that quantifies tumorigenicity by measuring a single cell’s ability to proliferate and form colonies in suspension within a semi-solid agarose gel [14]. As expected, 21 days after plating the cells on soft agar, cell transfected with miR-335 mimic formed significantly less colonies than cells transfected with NC (*p* = 0.001) or wild-type cells (Fig. 3c).

To explore if viability was altered, the MTS assay (which measures the redox state of the mitochondria) was used to indirectly assess the viability of cells transfected with either miR-335 mimic or inhibitor and their corresponding NCs. The results revealed that cells expressing miR-335 mimic showed a significant decrease in viability compared to those expressing mimic NC at 24 and 48 h post-transfection (*p* < 0.05) (Fig. 3d). Consequently, viability was significantly higher in cells expressing miR-335 inhibitor when compared with NC inhibitor and wild-type cells at 48 h (*p* < 0.01) (Fig. 3e). In addition, the transfected cells were assessed by the trypan blue dye exclusion assay. In accordance with the MTS assay, cells expressing high levels of miR-335 presented a lower viability percentage (83.6% ± 5.9; *p* < 0.001) compared to the control at 48 h after transfection (Fig. 3f). No statistically significant differences between wild-type cells and those transfected with corresponding NCs were observed in either the MTS or the trypan blue experiments. Taken together, all performed functional assays demonstrated the invasive and metastatic effect of miR-335 on AGS cell line.

### miR-335 alters tumor invasion, migration, and tumorigenicity in Hs 746T cell line

**Fig. 4:**
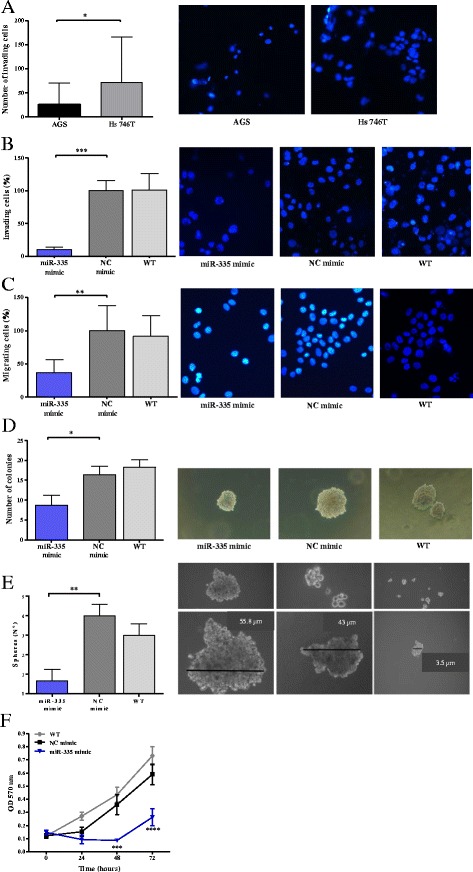
Increased expression levels of miR-335 inhibit cell invasion, cell migration, anchorage-independent cell growth, stemness properties, and cell viability in Hs 746T cells. **a** Comparison of invasive properties of AGS and Hs 746T cells. **b** Representative images for Hs 746T cells transfected with control NC/miR-335 mimic in invasion assay. **c** Hs 746T cells transfected with control NC/miR-335 mimic in migration assay. **d** Hs 746T cells transfected with control NC/miR-335 mimic in soft agar assay. Formation of colonies > 50 μm in diameter was scored at 2 weeks. **e** Hs 746T cells transfected with control NC/miR-335 mimic in spheroid formation assay. **f** Viability of Hs 746T cells transfected with control NC/miR-335 mimic in MTT assay. Results represent the means of three independent experiments, bars indicate SD. *р < 0.05, **p < 0.01, ***p < 0.001, ****р < 0.0001. WT, wild-type

To further confirm the role of miR-335 on tumor invasion, we chose the non-Asian gastric cancer cell line Hs 746T that has low expression of miR-335 (Additional file 1: Figure S1) and investigated its invasive properties. Using the Transwell inserts coated with Matrigel, we observed 2.8 times higher (*p* = 0.012) amount of Hs 746T cells that invaded Matrigel after 48 h when compared to AGS cells (Fig. 4a). To validate our presumption that the very low expression of miR-335 influences the high invasiveness of this metastatic cell line, we transfected these cells with miR-335 mimics to overexpress this miRNA and observed dramatic decrease of cells invading Matrigel (*p* < 0.001), (Fig. 4b).

To further validate our data obtained in AGS cell line, we repeated the functional assays in Hs 746T cells transfected with miR-335 mimic. After showing that these cells transfected with miR-335 mimic show significantly decreased invasive properties, we performed the migration assay and observed a diminution of cells overexpressing miR-335 migrated through the Transwell (37.1% ± 19.1; *p* < 0.01) (Fig. 4d). Anchorage-independent cell growth and stemness capacities by soft agar assay and spheroid formation assay, respectively (Fig. 4d, e), were also diminished in Hs 746T cells transfected with miR-335 mimic (*р* < 0.05 and *p* < 0.01, respectively). Finally, viability by MTT assay was also significantly diminished in Hs 746T transfected with miR-335 at 48 and 72 h post-transfection (*p* < 0.001) as shown in Fig. 4f.

In conclusion, metastasis-derived cell line Hs 746T cells express significantly less miR-335 and demonstrate higher invasive properties when compared to primary tumor-derived cell line AGS. In accordance with these findings, both cell lines transfected with miR-335 mimic show significantly decreased invasive and migratory properties, anchorage-independent cell growth capacities, and viability.

### miR-335 alters expression of genes involved in metastasis and tumor invasion

**Fig. 5:**
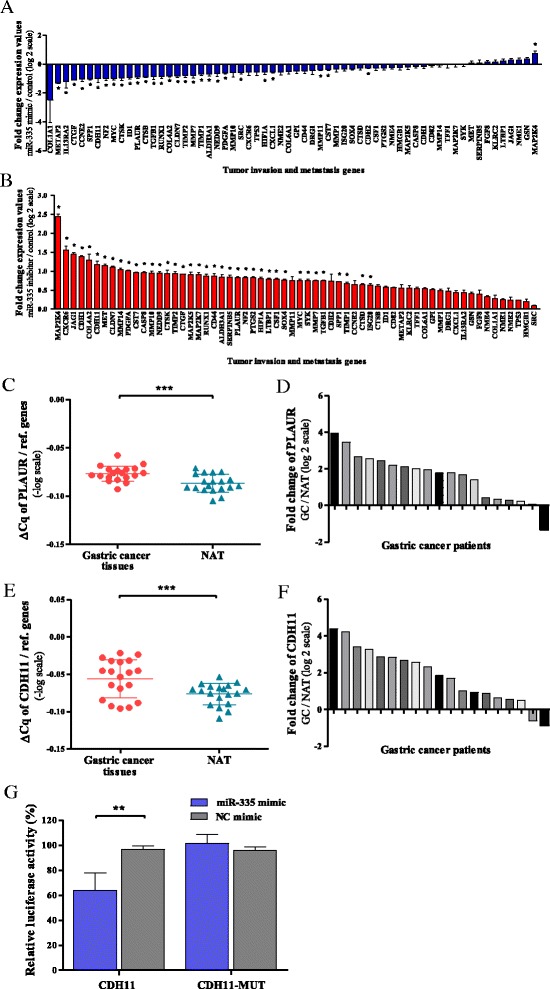
PCR array identifies metastasis and invasion downstream genes of miR-335. **a**, **b** Expression profiles of genes involved in metastasis and tumor invasion in AGS cells transfected with miR-335 mimic or with miR-335 inhibitor. Expression levels were normalized to three endogenous genes. Results indicate the mean values of three independent experiments ± SD. **c** ΔCq of PLAUR in 19 gastric tumor tissues compared to their matched non-tumor adjacent tissues (NAT) and normalized to reference genes. Data were transformed to logarithmic values (−log). **d** Relative expression of PLAUR in 19 samples of gastric tumor tissues, normalized to reference genes. **e** ΔCq of CDH11 in 19 gastric tumor tissues compared to NAT and normalized to reference genes. Data were transformed to logarithmic values (−log). **f** Relative expression of CHD11 in 19 samples of gastric tumor tissues, normalized to reference genes. Data were transformed to logarithmic values (log 2). **g** Direct interaction between miR-335 and CDH11 was detected by dual-luciferase reporter assay. Over-expression of miR-335 in AGS cells reduced the luciferase signal of CDH11 compared with NC mimic, while mutation of the miR-335-binding site diminished this suppressive effect. *р < 0.05, **p < 0.01

Due to generally low expression of miR-335 in gastric cancer cell lines, we chose the AGS cell line to overexpress and inhibit expression in order to identify downstream genes of miR-335 involved in gastric cancer progression. The mRNA expression profile of 62 genes involved in metastasis and tumor invasion pathway was analyzed by PCR array in cells transfected with miR-335 mimic or miR-335 inhibitor and their corresponding NCs. As shown in Fig. 5a, b, the presence of miR-335 mimic decreased the transcript expression levels of 19 (30.6%) (Table 2) of these genes, while the miR-335 inhibitor increased the same genes. Network enrichment analysis showed that nine of these genes (PLAUR, CDH11, COL4A2, CTGF, CTSK, MMP7, PDGFA, TIMP1, and TIMP2) mapped to known metastasis and cell invasion pathways (Additional file 2: Figure S2). In order to investigate the target genes of miR-335 that are implicated in metastasis and tumor invasion, we utilized the miRWalk database [15]. Searching for predicted and validated miR-335 target genes, we found that PLAUR (*p* = 0.046) and CDH11 (*p* = 0.049) are potential targets which participate in metastasis and tumor invasion pathway [16–18]. Moreover, Alfano et al. [19] had already demonstrated that miR-335 is a direct target of the 3′UTR of PLAUR and could interfere with the expression of the corresponding mRNA.

**Table 2:** Deregulated genes involved in metastasis and tumor invasion in cells transfected with miR-335 mimic and inhibitor

Symbol	Name	Gene function	*p* value overexpressed miR-335 cells	*p* value inhibited miR-335 cells	References
ALDH3A1	Aldehyde dehydrogenase 3 family member A1	Oncogene	0.029	0.029	[41]
CDH11	Cadherin 11	Oncogene Tumor suppressor	0.029	0.029	[42, 43]
CLDN7	Claudin 7	Oncogene	0.029	0.029	[44]
COL4A2	Collagen type IV alpha 2	Oncogene	0.029	0.029	[45]
CST7	Cystatin F	Oncogene	0.029	0.029	[46]
CTGF	Connective tissue growth factor	Oncogene Tumor suppressor	0.029	0.027	[47, 48]
CTSK	Cathepsin K	Oncogene	0.029	0.029	[49]
HIF1A	Hypoxia inducible factor 1 alpha subunit	Oncogene	0.042	0.029	[50]
MMP7	Matrix metallopeptidase 7	Oncogene	0.029	0.029	[51]
MYC	V-myc avian myelocytomatosis viral oncogene homolog	Oncogene	0.029	0.029	[52]
NEDD9	Neural precursor cell expressed, developmentally downregulated 9	Oncogene	0.029	0.029	[53]
NF2	Neurofibromin 2 (merlin)	Oncogene Tumor suppressor	0.029	0.029	[54, 55]
PDGFA	Platelet-derived growth factor subunit A	Oncogene	0.029	0.029	[56]
PLAUR	Plasminogen activator, urokinase receptor	Oncogene	0.029	0.029	[57]
RUNX1	Runt-related transcription factor 1	Tumor suppressor Oncogene	0.029	0.029	[58, 59]
SPP1	Secreted phosphoprotein 1	Oncogene	0.029	0.027	[60]
TGFB1	Transforming growth factor beta 1	Oncogene	0.029	0.029	[61]
TIMP1	TIMP metallopeptidase inhibitor 1	Tumor suppressor Oncogene	0.029	0.029	[62]
TIMP2	TIMP metallopeptidase inhibitor 2	Tumor suppressor Oncogene	0.029	0.029	[63, 64]

Mann-Whitney *U* test

### PLAUR and CDH11 genes are overexpressed in gastric cancer clinical samples

Based on binding with PLAUR, we further validated the expression of PLAUR in tumors with low expression of miR-335 (less than median of the total samples). As shown in Fig. 5c, we observed elevated levels of PLAUR when compared with their paired NAT (*p* = 0.001). By plotting the relative expression of PLAUR in each of the separate samples, it was observed that 18/19 (94.7%) patients had increased PLAUR (Fig. 5d). In the same clinical samples, expression of CDH11 was validated. Figure 5e shows similar increase of CDH11 expression in tumor samples (*p* = 0.0006), and when plotting these relative expressions, 17/19 (89.5%) patients demonstrated increased CDH11 mRNA levels (Fig. 5d).

### The CDH11 3'UTR is the direct target for miR-335  

To determine if CDH11 is a direct target of miR-335, we cloned a fragment of the 3′UTR of the CDH11 gene into a dual-luciferase construct pmirGLO. Notably, the 3′UTR of CDH11 appeared to be repressed by miR-335 by 36% ± 14 in AGS cells compared with the NC (*p* = 0.005) (Fig. 5g). When mutant fragment of the 3′UTR of the CDH11 gene was cloned, no repression was observed. These results indicate that the miR-335 directly targets the 3′UTR of CDH11.

### Correlation between MEST and miR-335 expression

The miR-335 locus resides in the second intron of the mesoderm-specific transcript (MEST) gene from which its miRNA is processed [20]. Therefore, we surveyed the expression levels of miR-335 and MEST across 386 clinical samples from The Cancer Genome Atlas (TCGA). This analysis uncovered a strong correlation (Spearman’s rank correlation test, *r* = 0.83; *p* = 0.0001) between miR-335 and the gene from which it arises. These findings were validated in AGS and Hs 746T cell lines (Additional file 3: Figure S3). Taken together, our survey supports the notion that miR-335 is co-expressed with MEST, under the control of the promoter methylation of its host gene.

### miR-335 expression is downregulated in plasma from gastric cancer patients and associated with DNA methylation of the promoter region of MEST gene

**Fig. 6:**
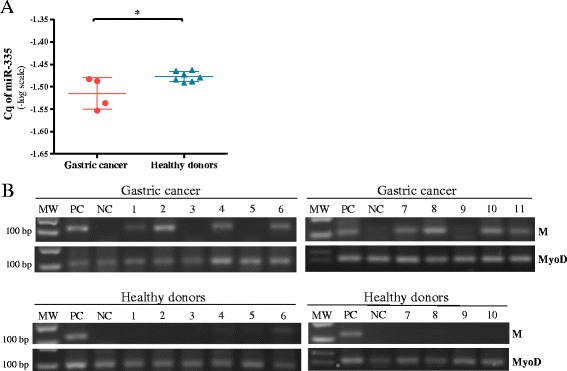
Expression of miR-335 and DNA methylation of the promoter region of MEST gene in plasma from gastric cancer patients and healthy donors. **a** Cq of miR-335 expression among four gastric cancer plasma samples and seven plasma samples from healthy donors. Data were transformed to logarithmic values (−log). Results indicate the mean ± SD. **b** Illustrative results of MSP in gastric cancer and healthy donor’s plasma samples. MyoD was used as a control of DNA conversion. MW, weight marker; M, PCR product with primers specific for methylated promotor region of miR-335 host gene (MEST); PC, positive control of methylation (methylated gastric cancer cell line); NC, negative control of methylation (peripheral blood lymphocytes)

The relationship between the expression of miR-335 and aberrant promoter methylation of its host gene (MEST) has been shown [21, 22]. Thus, we surveyed this relationship in plasma samples from gastric cancer cases and healthy donors (*N* = 11) to found a significant difference in the expression of miR-335 between these two groups (*p* < 0.05) (Fig. 6a). To explore the role of aberrant hypermethylation as a surrogate biomarker for non-invasive diagnosis of gastric cancer, we performed methylation-specific PCR (MSP) assay [21] in plasma samples from 41 gastric cancer and 30 healthy donors. Positive bands were observed in 23 (56.1%) out of 41 plasma samples from gastric cancer cases but only in 9 (30%) out of 30 plasma samples from healthy donors. This correlation difference was significant (*Z* = 1.96, *p* = 0.029, Pearson’s correlation) (Fig. 6b).

## Discussion

The involvement of miR-335 in gastric cancer progression remains controversial. Among five published clinical studies to date, only one of them has shown downregulation of miR-335 in gastric cancer samples [23] while three showed no significant differences between tumor and non-tumor tissues [12, 17, 21], and one study reported overexpression of miR-335 in gastric cancer patients with recurrence disease [11]. Each of these reports was compiled from Asian patients’ cohorts; however, geographical and ethnic variables are known to influence gene expression [24, 25]. Therefore, a study investigating the expression of this miRNA in other geographical locations could contribute to clarify these controversies. Herein, in an Amerindian/Hispanic cohort, we report that downregulation of miR-335 in gastric cancer tissues in respect to their NAT is associated with depth of tumor invasion and lymph node metastasis. In addition, downregulation of miR-335 is a prognostic factor for overall survival in Amerindian/Hispanic gastric cancer patients.

To clarify the biological role of miR-335 in gastric cancer, we performed in vitro functional studies in the AGS and Hs 746T gastric cancer cell lines, two of the widely used models for understanding the molecular mechanisms involved in the progression of gastric cancer [26]. Our results showed that exogenous miR-335 expression was able to inhibit migration, invasion, tumorigenicity, and viability of these cells. In accordance, inhibition of miR-335 expression promoted these processes. Our results on invasion and cell viability are consistent with, and complement, earlier studies in other cell lines [12, 23]. Moreover, our functional assays of migration and tumorigenicity further support the potential role of miR-335 as a tumor suppressor gene in gastric cancer.

Genes downstream of miR-335 in metastasis and tumor invasion have not yet been completely characterized in gastric cancer. Xu et al. [12] found that miR-335 might function as a metastasis suppressor by targeting Bcl-w and specificity protein 1 (Sp1), and Yang et al. [23] has reported that miR-335 regulates growth and apoptosis by direct targeting of survivin. Another study showed that exogenous miR-335 expression repressed expression of RASA1, which has been reported to play role in cancer cell invasion [21]. Herein, we comprehensively evaluated the expression of genes involved in the metastasis and tumor invasion pathways. We identified that up to 19 out of 62 (30.6%) genes were significantly associated with changes in expression levels of miR-335. These genes belong to relevant intracellular signaling pathways in cancer such as PI3K-Akt (COL4A2, MYC, PDGFA, SPP1), proteoglycans (HIF1A, MYC, PLAUR, TGFB1), and Hippo (CTGF, MYC, NF2, TGFB1). As a single miRNA can target several messenger RNAs, dysregulation of miRNAs can effectively affect multiple signaling pathways leading to tumor formation and metastasis [27]. In this scenario, Ingenuity Pathway Analysis (IPA) narrowed these 19 genes to 9 genes that mapped on a network leading to metastasis and cell invasion. Among these genes, PLAUR, a validated target of miR-335 [19], significantly increased mRNA levels after knockdown of miR-335 expression in gastric cancer cells. PLAUR is membrane-bound glycoprotein with a GPI anchor that encodes the receptor of urokinase-type plasminogen activator [28]. PLAUR binds and activates PLAU, a serine protease capable of converting plasminogen to active plasmin, which degrades components of the extracellular matrix, thus facilitating invasion and metastasis [29]. Furthermore, PLAUR has signaling properties through interactions with membrane-bound integrins, which are able to affect migration and cell proliferation [30]. In gastric cancer, the overexpression of PLAUR has been reported to be closely related to cell invasion and metastasis [31, 32]. Our in vitro analysis also showed a significant increase of PLAUR expression in miR-335 knockdown cells and, consequently cells overexpressing miR-335 exhibited low level of PLAUR. Accordingly, elevated levels of PLAUR were observed in tumor tissues when compared with their paired non-tumor mucosa. The other significant gene, CDH11, encodes a type II classical cadherin, an integral membrane protein that mediates calcium-dependent cell-cell adhesion [33]. CDH11 has been reported deregulated in various tumor types, suggesting a role in tumor invasion [16, 34], and its overexpression was found in advanced gastric cancer [18]. Ultimately, we identified CDH11 as a direct target of miR-335.

The strong correlation between the expression of miR-335 and MEST, the gene from which it arises, suggests that the mechanisms responsible for silencing miR-335 expression should be the promoter methylation of its host gene [21, 22]. Thus, we evaluated if the methylation of the promoter of the MEST gene [21] could be detected in plasma as a useful approach to non-invasive assessment of gastric cancer. DNA methylation in plasma has been proposed as a biomarker for risk prediction, prognostication, and prediction of treatment response in gastric cancer [21, 35]. In addition, several studies have shown that downregulation of microRNAs is associated with DNA methylation of the promoter region of its host genes [25, 36]. Envisaging the clinical application of the down-regulated of miR-335, we expand to plasma the reported inverse correlation between expression and DNA methylation in gastric cancer tissues and cell lines [21]. Herein, we not only demonstrated that inverse correlation occurs in plasma, but also that significant difference between gastric cancer patients and healthy donors were observed. Thus, our findings support the idea that DNA methylation of miR-335 may be a promissory strategy for non-invasive approach to gastric cancer.

## Conclusions

In summary, we favor the role of tumor suppressor gene of miR-335 by clinical and in vitro functional assays. Our comprehensive evaluation of metastasis and invasion pathway led us to identify and confirm that PLAUR, a validated target of miR-335, and CDH11 are overexpressed in gastric cancer tissues. In addition, CDH11 was identified as a direct target of miR-335. The inverse correlation between miR-335 expression and methylation in plasma not only expand previous findings regarding the epigenetic regulation of miR-335 but also propose that methylation of the promoter region of miR-335 might be a novel candidate for non-invasive diagnosis of gastric cancer. Further research in independent cohorts will be required to develop this clinical application.

## Methods

### Clinical samples

Thirty-eight gastric tumor tissues and their pair-matched adjacent non-tumor tissues (that were a minimum of 10 cm from tumor lesion) were obtained with written informed consent from patients undergoing total gastrectomy at Hernán Henriquez Aravena Hospital, Temuco or at Sótero del Río Hospital, Santiago, Chile between the years 2008 and 2015. Fresh samples were immediately frozen after its resection and stored at − 80 °C by Tissue Biobank of Universidad de La Frontera, Temuco, Chile or directly processed in the Laboratory of Oncology at Pontificia Universidad Católica de Chile, Santiago. All patients were stratified according to the AJCC gastric cancer staging system [13]. Clinicopathologic dates were taken from medical records. Plasma samples were obtained from Centro de Referencia de Salud San Rafael, Santiago and Sótero del Río Hospital, Santiago, Chile.

### Gastric cancer cell lines and culture conditions

AGS and Hs 746T gastric carcinoma cell lines were purchased from American Type Culture Collection (ATCC, Manassas, VA, USA). AGS cells were grown in RPMI 1640 medium and Hs 746T cells in DMEM medium, both supplemented with 10% heat-inactivated fetal bovine serum (FBS), 10 units/ml penicillin, and 10 mg/ml streptomycin at 37 °C in a humidified atmosphere containing 5% CO_2_ and subcultured during the logarithmic phase. AGS cells were verified by short tandem repeat DNA profiling analysis.

### RNA extraction and RT-qPCR of miR-335

Total RNA was extracted from gastric cancer tissues and paired NAT fresh frozen cases using the mirVana miRNA Extraction kit (Ambion, Life Technologies, USA), according to the manufacturer’s protocol. Total RNA from cell lines was isolated using TRIzol (Thermo Fisher Scientific) according to the manufacturer’s instructions including the adding of 20 μg glycogen (Roche) prior to isopropyl precipitation. Pellet was dissolved in 20 μl nuclease-free water. For RNA isolation of plasma 0.75 ml TRIzol LS (Thermo Fisher Scientific) was added to 0.25 ml plasma sample and further processed according to the manufacturer’s protocol and as described above.

miR-335 was quantitated by real-time PCR using TaqMan® Small Assays human (Life Technologies, Foster City, CA), following the protocol provided by the manufacturer. Briefly, mature miRNA was reverse transcribed from 10 ng of RNA using TaqMan® MicroRNA Reverse Transcription kit (Applied Biosystems, Life Technologies, USA) following the protocol provided by the manufacturer. qPCR was carried out using TaqMan® Universal PCR Master Mix with LightCycler® 480 Instrument (Roche Diagnostics GmbH, Roche Applied Science, Germany) according to manufacturer’s protocol. Small RNA RNU6B was used as internal control for input normalization. Primers and probes were obtained as predesigned TaqMan® Small Assays human (miR-335 Assay ID 000546, RNU6B ID 001093). The threshold cycle (Ct) was determined using the LightCycler software release 1.5.0. The relative miRNA expression was expressed as 2^−ΔΔCT^ [37].

### Oligonucleotide transfection

Lipofectamine® 2000 reagent (Invitrogen, Life Technologies, USA) was used to deliver single-stranded RNA molecules (mirVana™ miRNA Mimics and Inhibitors; Ambion, Life Technologies, USA) into AGS cells or Hs 746T as per manufacturer’s instructions. Briefly, cells were seeded at 2 × 10^5^ cells per well of a 6-well plate and transfected with 30 nM of mimic or inhibitor of hsa-miR-335-5p (mature sequence 5′- UCAAGAGCAAUAACGAAAAAUU -3′, miRBase accession number: MI0000816) [38] when reached 70% confluence. Thirty nanomolar of random sequence of miRNA mimic or inhibitor molecules (mirVana™ miRNA Mimic and Inhibitor Negative Controls; Ambion, Life Technologies, USA) were used as negative controls. After transfection, cells were added to pre-warmed media and immediately placed in an incubator at 37 °C in a 5% CO_2_ atmosphere. Cells were harvested at 24 and 48 h after transfection for functional assays. Transfection efficiency was evaluated by RT-qPCR as described above Additional file 4: Figure S4).

### PCR array

For PCR array, AGS cell transfected with NC/miR-335 mimic or with NC/miR-335, inhibitor was used. The Pathway PCR array was purchased from Real Time Primers (Elkins Park, PA, USA), contained 88 primer sets directed against tumor invasion/metastasis genes and 8 housekeeping gene primer sets provided in a 96-well microplate (10 μM). PCR array library was used according to the manufacturer’s specifications (Real Time Primers, PA, USA). Normalization was performed with three reference genes RPL13A, ACTB, and PP1A, previously chosen by geNorm v3.5 software [39].

### RT-qPCR

The extracted RNA was treated with DNase (Promega) according to the manufacture’s protocol. Reverse transcription (RT-PCR) was performed with 1000 ng of DNase-treated RNA using the reverse transcriptase enzyme M-MLV (Promega). The qPCRs were performed using a Brilliant II SYBR Green QPCR Master Mix (Agilent) following the protocol provided by the manufacturer. Briefly, 20 μl reaction volumes in 96-well plates were analyzed in LightCycler 480 (Roche Diagnostics) under the following conditions: initial 10 min denaturation at 95 °C, followed by 45 cycles at 95 °C for 15 s, and 60 °C for 60 s. Following primer pairs were used for cDNA amplification: CDH11 (forward 5′- AGAGAGCCCAGTACACGTTGA -3′, reverse 5′- TTGGCATGATAGGTCTCGTGC -3′), PLAUR (forward 5′- TGTAAGACCAACGGGGATTGC -3′, reverse 5′- AGCCAGTCCGATAGCTCAGG -3′), and MEST (forward 5′- CGCAGGATCAACTTCTTTC -3′, reverse 5′- CATCAGTCGTGTGAGGATGG -3′). Normalization was performed as above. The qPCRs were performed in triplicates.

### Plasmid construction

A 355-bp fragment of the CDH11-3′UTR and a 253-bp fragment of mutant CDH11-3′UTR were amplified by PCR from human genomic DNA. For CDH11, the following sense (5′- AAA GAGCTCGGAGAAGTCTAACGCTGA -3′) and antisense (5′- TGCCCTGCAGG CGTCTCTCACTGAAACAAG -3′) primers and, for mutant, the following sense (5′- CCGA GAGCTCCGCTGAACTGACAATGAAG -3′) and antisense (5′- ATACCTGCAGG CAAGATTGATGCTCAACCAC -3′) primers were used. The PCR products were cloned into a pmirGLO Dual-Luciferase miRNA Target Expression Vector (Promega) where SacI and SbfI (both Thermo Scientific) sites were introduced into the multiple cloning sites. Both constructs were confirmed by DNA sequence analysis.

### Luciferase assay

AGS cells were plated in 96-well plates (2 × 10^4^/well). After 24 h, cells were co-transfected with 60 ng of pmirGLO-3′UTR/CDH11 construct or with mutant pmirGLO-3′UTR/CDH11 construct and with 30 nM of miR-335 mimic or NC mimic (both sequence reported above) using FuGENE HD (Promega) transfection reagent. Twenty-four-hour post-transfection, the firefly and Renilla luciferase activities were measured with a luminometer using the dual-luciferase assay system (Promega), according to the manufacturer’s instructions. Assay was repeated three times, and for each sample, firefly luciferase activity was normalized to Renilla luciferase expression.

### Cell migration and invasion assay

For the migration assay, 5 × 10^4^ AGS cells in serum-free media were placed into the upper chamber of an insert with 8 μm pore size polycarbonate membrane (Corning, NY, USA). For the invasion assay, the Transwell insert was coated with 20 μg Matrigel (Corning, NY, USA), and 7 × 10^4^ AGS cells were plated onto the top of the coated filters. The cells were treated with corresponding oligonucleotides 24 h before starting the assay. The medium containing 10% FBS was placed in the lower chamber to act as a chemoattractant. After 18 h of incubation, the cells that did not migrate or invade through the pores were removed with a cotton swab and the membranes were fixed with methanol and stained with 0.1% crystal violet in 25% methanol/PBS. Five visual fields from each membrane were randomly selected and the number of cells that had migrated was counted using an optical microscope (Zeiss Scope A.1 Axio, 10×). For the invasions of Hs 746T and AGS cells in Fig. 4, the protocol was modified as follows: 5 × 10^4^ cells were left to invade Matrigel for 48 h, fixed with 3.8% PFA, stained with DAPI, and 9 pictures per membrane were taken with fluorescent microscope (Olympus BX53F, 20×). The values of migration and invasion were measured with ImageJ® v1.48r program (NIH, USA). Invasive and migratory ability is expressed as the mean of cells per field.

### Wound healing assay

Cells, transfected 24 h prior the assay, were seeded in 6-well plates, and a thin line was scratched into the monolayer of cells with a pipette tip simulating a wound. Zero, 10, and 20 h after the scratch, the width of the wound was evaluated under inverted microscope to assess the migration ability of the cells. Four representative photographs were taken, and the area of the cut was quantified with v1.48r ImageJ® software.

### Cell viability assay

To analyze the real-time cell growth, MTS colorimetric assay (CellTiter 96® AQueous One Solution Cell Proliferation Assay, Promega, USA) was used following the manufacturer’s instructions. Twenty-four hours after transfection, the AGS cells were trypsinized and seeded at a density of 8 × 10^3^ cells/well in 96-well plates in complete medium and incubated at 37 °C for 12, 24, and 48 h. After, the MTS reagent was added into each well and incubated at 37 °C for 2 h. Absorbance was read at 492 nm using a microplate reader (Bio-Rad, Hercules, CA, USA).

### Trypan blue dye exclusion assay

Twenty-four hours after transfection cells were trypsinized and seeded at a density of 4 × 10^4^ cells/well in 24-well plates in complete medium and incubated at 37 °C. After 12, 24, and 48 h, cell suspensions were prepared with 0.4% trypan blue solution, and the counting of viable and nonviable cells was performed in a Neubauer chamber.

### Clonogenic capacity

Two hundred cells pretreated with miRNA mimic and inhibitor were plated in complete medium in each well of a 6-well plate and incubated at 37 °C for 14 days. Colonies were subsequently fixed and stained with 0.5% crystal violet in 25% methanol/PBS. Digital images were obtained by scanning at high resolution, and surviving colonies (≥ 50 cells per colony) were counted using the ImageJ® v1.48r program.

### Soft agar colony formation assay

Anchorage-independent cell growth was determined by colony formation in soft agar. Twenty-four hours after transfection, 2.5 × 10^3^ cells were mixed with 0.35% agarose (UltraPure™ LMP Agarose; Invitrogen) in RPMI medium and quickly plated on top of a solidified layer of 1% agarose in growth medium in a 6-well plate. Solidification was completed at room temperature for 45 min. Every 3 days, growth medium was changed, and after 14–21 days, the colony formation of cells was evaluated. All colonies larger than 50 μm were counted in each well. The experiment was repeated three times.

### Sphere formation asssay

To determine stemness, 2 × 10^3^ cells were seeded into 2% agarose-coated 12-well plates 24 h post-transfection with miR-335 mimic or NC. Spheres > 40 μm in diameter were scored at 10–12 days.

### DNA extraction and conversion

DNA from 0.5–1 ml plasma from 41 gastric cancer patients and 30 healthy donors was extracted with QIAamp DNA Mini kit (QIAGEN, USA) according to manufacturer’s instructions. Extracted DNA was dissolved in 100 μl and subjected to bisulfite conversion and purification using the EZ DNA Methylation-GoldTM kit (Zymo Research, USA) according to the manufacturer’s protocol. The methylation-specific PCR (MSP) was performed as previously described in Li et al. [21]. Based on this paper, primers amplifying the second CpG island were used (forward 5′- GGTTTTAAAAGTCGGTGTTTATTC -3′, reverse 5′- AACTACAACCACTCCGACGTA -3′).

### Network analysis of tumor invasion/metastasis genes

Log ratios of significantly overexpressed genes from our PCR array by inhibition of miR-335 were loaded into IPA (Ingenuity Systems®, QIAGEN, USA). IPA identified significant networks by computing a score for each network derived from the *p* value for observed vs expected overlap of genes from our dataset that map to a particular network [40]. MAP tool was applied to show the activation or inhibition of canonical pathways and biological networks in silico.

### Statistical analysis

Statistical analyses were performed using SPSS for Windowsv.17.0 (SPSS, Chicago, IL) and GraphPad Prism 5.0 (GraphPad Software Inc., CA, USA). Association with clinicopathological variables was examined using Mann-Whitney test. Kaplan-Meier survival curves were plotted and compared using the log-rank test. The results of functional experiments were evaluated using the one-way or two-way ANOVA with the corresponding post-test. The results of PCR array analysis were measured using a Mann-Whitney U test. For miRNA and gene expression analysis, Student’s *t* test for paired samples was used for mean comparison between the two groups (NAT versus tumors). A two-tailed *p* < 0.05 was considered statistically significant. All the statistics were expressed as mean ± standard deviation (SD) of three independent experiments. A point-biserial correlation was run to determine the relationship between gastric cancer and healthy donor’s plasma samples.

## Additional files

Additional file 1: Figure S1. ΔCq of miR-335 in non-Asian gastric cancer cell lines. miR-335 was normalized by RNU6B. Data were transformed to logarithmic values (−log). Results indicate the mean ± SD (PPTX 56 kb)

Additional file 2: Figure S2. Ingenuity Pathway Analysis (IPA) for network enrichment analysis identified metastasis and invasion downstream genes of miR-335. Network of nine significantly overexpressed (red) genes during miR-335 inhibition. MAP tool shows activation and inhibition of neighboring genes and predicts activation of metastasis and invasion of cells in silico. *p < 0.05 (PPTX 253 kb)

Additional file 3: Figure S3. miR-335 expression depends on the expression of its host gene, mesoderm specific transcript (MEST). **A** miR-335 log2-expression levels are shown in the y-axis. A linear model has been fit (blue regression line) using RNAseq data from 368 tumor samples from the stomach adenocarcinoma The Cancer Genome Atlas (TCGA) consortium; according to the model: log2(miR-335) = 0.8249*log2(MEST), *p* = 2E^−16^, *N* = 368. **B** High and low miR-335 and MEST linear co-expression in AGS and Hs 746T cell lines (PPTX 129 kb)

Additional file 4: Figure S4. Transfection efficiency of human gastric cancer AGS and Hs 746T cell lines treated with miR-335 mimics/inhibitor. Increased or decreased expression of miR-335 in AGS and Hs 746T transfected with NC/miR-335 mimic or with NC/miR-335 inhibitor. Expression of miR-335 was normalized to RNU6B. Data were transformed to logarithmic values (log 2) (PPTX 51 kb)
