# Correction to: MicroRNA-335-5p is a potential suppressor of metastasis and invasion in gastric cancer

**DOI:** 10.1186/s13148-021-01036-2

**Published:** 2021-03-08

**Authors:** Alejandra Sandoval-Bórquez, Iva Polakovicova, Nicolás Carrasco-Véliz, Lorena Lobos-González, Ismael Riquelme, Gonzalo Carrasco-Avino, Carolina Bizama, Enrique Norero, Gareth I. Owen, Juan C. Roa, Alejandro H. Corvalán

**Affiliations:** 1 Advanced Center for Chronic Diseases (ACCDiS), Pontificia Universidad Católica de Chile, Santiago, Chile; 2 Laboratory of Molecular Pathology, Department of Pathology, School of Medicine, BIOREN-CEGIN, and Graduate Program in Applied Cell and Molecular Biology, Universidad de La Frontera, Temuco, Chile; 3 Center UC for Investigational in Oncology (CITO), Pontificia Universidad Católica de Chile, Santiago, Chile; 4 Instituto de Química, Faculty of Science, Pontificia Universidad Católica de Valparaíso, Valparaiso, Chile; 5 Advanced Center for Chronic Diseases (ACCDiS), Universidad de Chile, Santiago, Chile; 6 Fundación Ciencia Y Vida, Parque Biotecnológico, Santiago, Chile; 7 Department of Pathology, Faculty of Medicine, Hospital Clínico Universidad de Chile, Santiago, Chile; 8 Department of Pathology, Faculty of Medicine, Pontificia Universidad Católica de Chile, Santiago, Chile; 9 Esophagogastric Surgery Unit, Hospital Dr. Sótero del Río, Santiago, Chile; 10 Digestive Surgery Department, Pontificia Universidad Católica de Chile, Santiago, Chile; 11 Department of Physiology, Faculty of Biological Sciences, Pontificia Universidad Católica de Chile, Santiago, Chile; 12 Department of Hematology-Oncology, Faculty of Medicine, Pontificia Universidad Católica de Chile, Santiago, Chile

## Correction to: Clinical Epigenetics (2017) 9:114 https://doi.org/10.1186/s13148-017-0413-8

Following publication of the original article [1], the authors identified an error in Fig. 2. The correct figure is given below (Fig. 2).

**Fig. 2:**
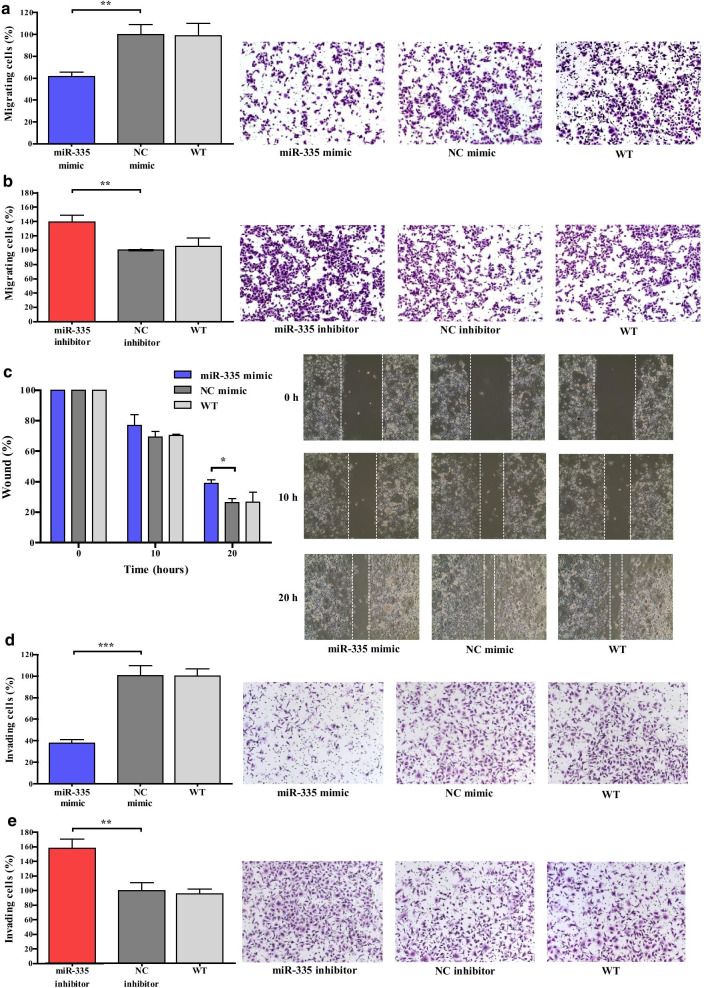
Overexpression of miR-335 inhibits cell migration and invasion. **a**, **b** Representative images of AGS cells transfected with NC/miR-335 mimic or with NC/miR-335 inhibitor in migration assay. **c** Representative images of AGS cells transfected with control NC/miR-335 mimic in wound healing assay. **d**, **e** Representative images of AGS cells transfected with control NC/miR-335 mimic or with NC/miR-335 inhibitor in invasion assay. Results represent the means of three independent experiments; bars indicate SD. *p < 0.05, **p < 0.01, ***p < 0.001. WT, wild type
