# Supplementary figures and images for: MicroRNA-335-5p is a potential suppressor of metastasis and invasion in gastric cancer
Source: Clin Epigenetics. 2017 Oct 17;9:114. doi: 10.1186/s13148-017-0413-8 (PMC5645854; doi:10.1186/s13148-017-0413-8)

## Slide 1
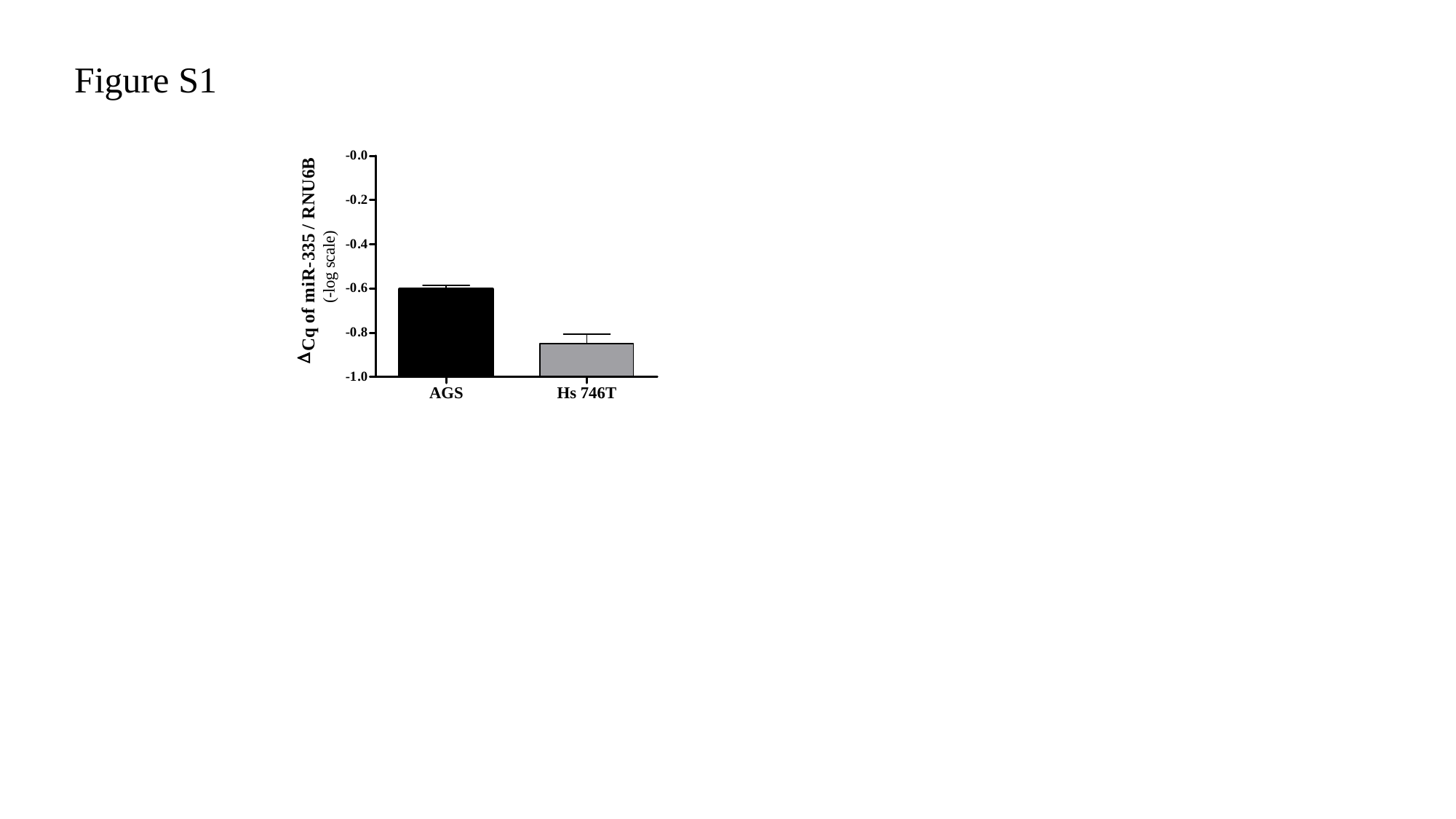

Figure S1

Supplement: Supplementary file 1 — ΔCq of miR-335 in non-Asian gastric cancer cell lines. miR-335 was normalized by RNU6B. Data were transformed to logarithmic values (−log). Results indicate the mean ± SD (PPTX 56 kb) [file 13148_2017_413_MOESM1_ESM.pptx]

## Slide 1
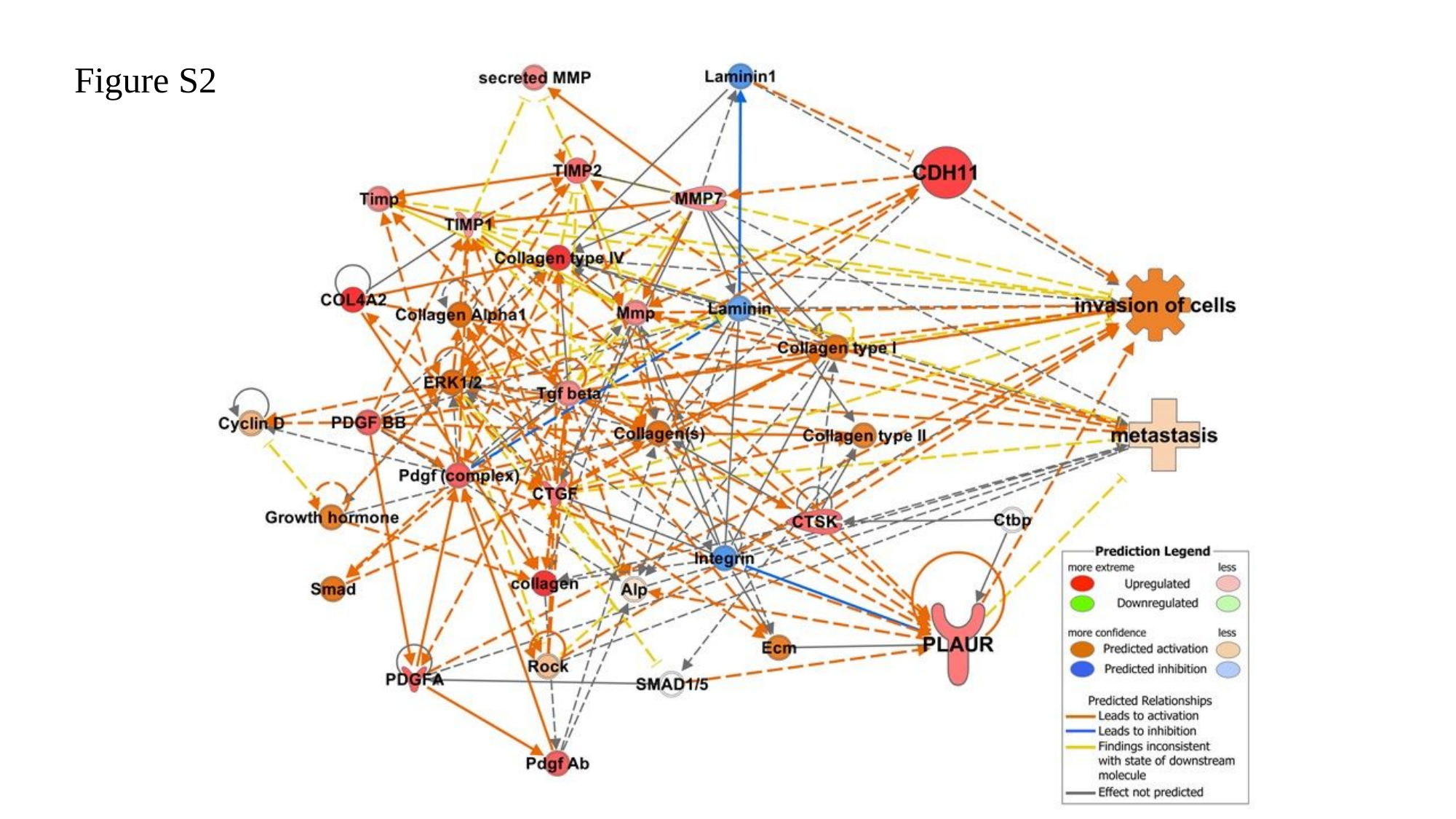

Figure S2

Supplement: Supplementary file 2 — Ingenuity Pathway Analysis (IPA) for network enrichment analysis identified metastasis and invasion downstream genes of miR-335. Network of nine significantly overexpressed (red) genes during miR-335 inhibition. MAP tool shows activation and inhibition of neighboring genes and predicts activation of metastasis and invasion of cells in silico. *p < 0.05 (PPTX 253 kb) [file 13148_2017_413_MOESM2_ESM.pptx]

## Slide 1
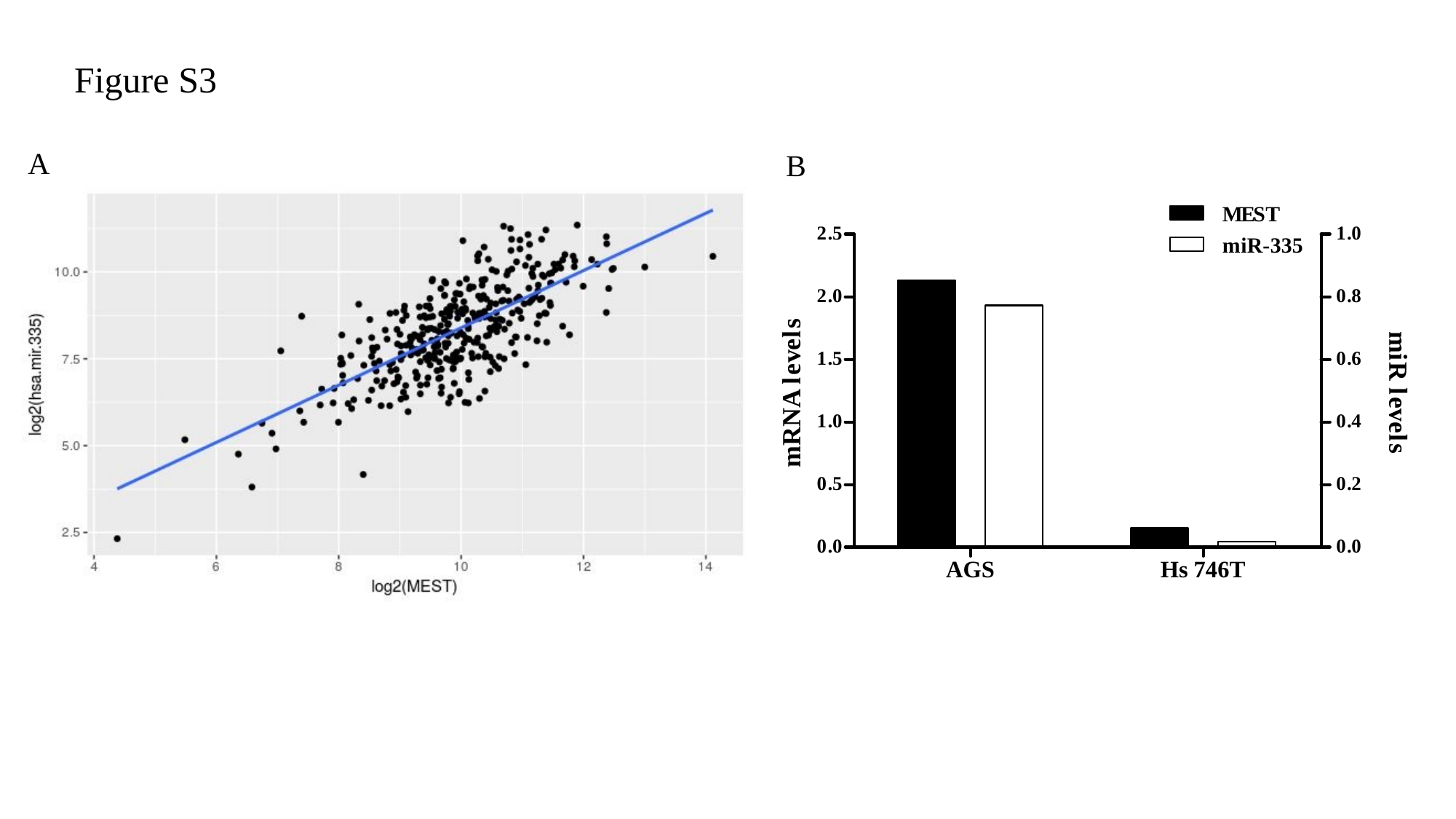

Figure S3
A
B

Supplement: Supplementary file 3 — miR-335 expression depends on the expression of its host gene, mesoderm specific transcript (MEST). A miR-335 log2-expression levels are shown in the y-axis. A linear model has been fit (blue regression line) using RNAseq data from 368 tumor samples from the stomach adenocarcinoma The Cancer Genome Atlas (TCGA) consortium; according to the model: log2(miR-335) = 0.8249*log2(MEST), p = 2E−16, N = 368. B High and low miR-335 and MEST linear co-expression in AGS and Hs 746T cell lines (PPTX 129 kb) [file 13148_2017_413_MOESM3_ESM.pptx]

## Slide 1
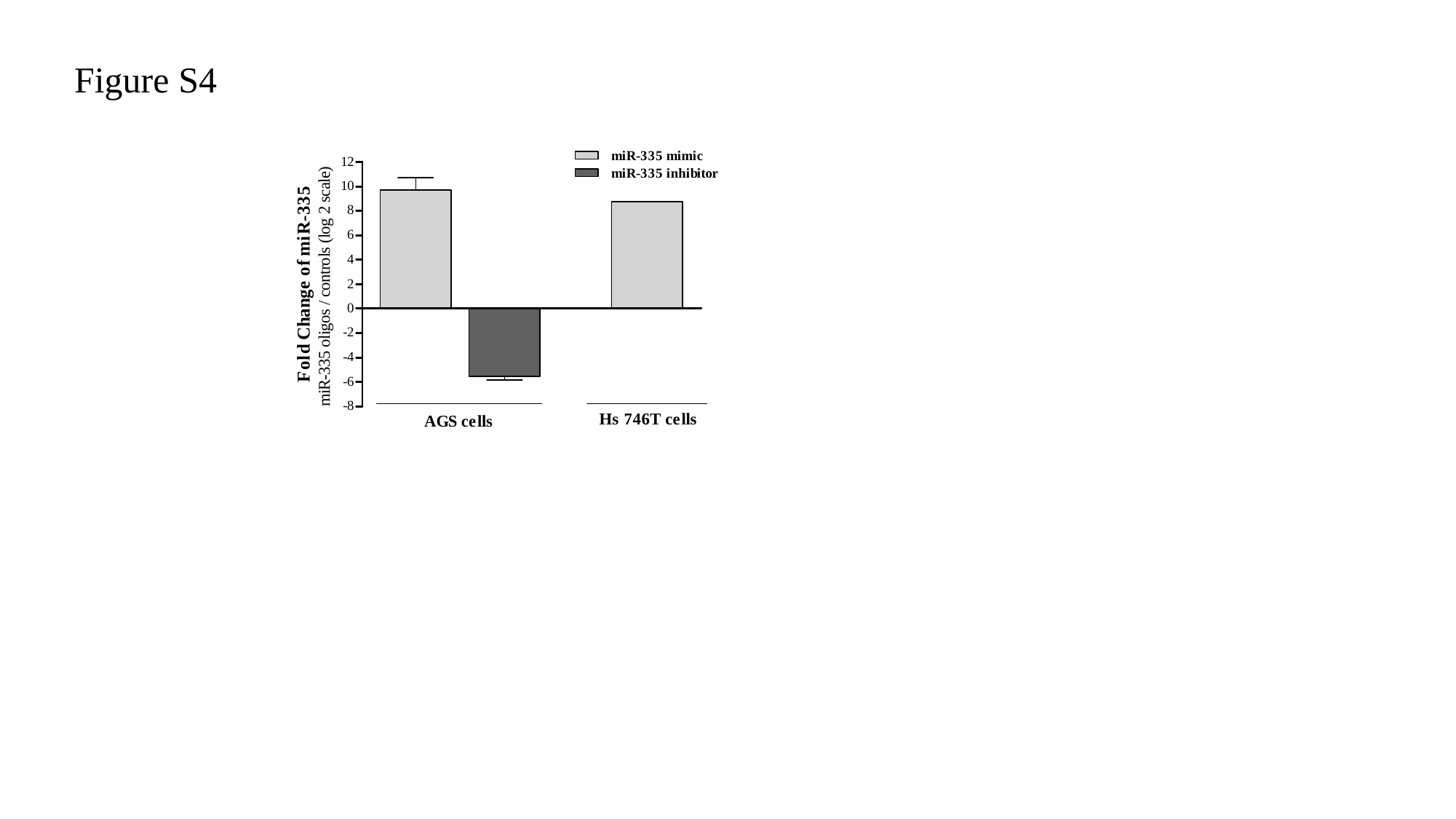

Figure S4

Supplement: Supplementary file 4 — Transfection efficiency of human gastric cancer AGS and Hs 746T cell lines treated with miR-335 mimics/inhibitor. Increased or decreased expression of miR-335 in AGS and Hs 746T transfected with NC/miR-335 mimic or with NC/miR-335 inhibitor. Expression of miR-335 was normalized to RNU6B. Data were transformed to logarithmic values (log 2) (PPTX 51 kb) [file 13148_2017_413_MOESM4_ESM.pptx]
